# Alterations in heart rate variability in patients with peripheral arterial disease requiring surgical revascularization have limited association with postoperative major adverse cardiovascular and cerebrovascular events

**DOI:** 10.1371/journal.pone.0203519

**Published:** 2018-09-13

**Authors:** Karri T. Utriainen, Juhani K. Airaksinen, Olli J. Polo, Harry Scheinin, Ruut M. Laitio, Kari A. Leino, Tero J. Vahlberg, Tom A. Kuusela, Timo T. Laitio

**Affiliations:** 1 Division of Medicine, Turku University Hospital, Turku, Finland; 2 Sleep Research Centre, University of Turku, Turku, Finland; 3 Heart Centre, Turku University Hospital and University of Turku, Turku, Finland; 4 Department of Pulmonology, Tampere University Hospital, Tampere, Finland; 5 Division of Perioperative Services, Intensive Care Medicine and Pain Management, Turku University Hospital, Turku, Finland; 6 Department of Biostatistics, University of Turku, Turku, Finland; 7 Department of Physics and Astronomy, University of Turku, Turku, Finland; Charité - Universitätsmedizin Berlin, GERMANY

## Abstract

**Objective:**

Obstructive sleep apnea (OSA) is common in peripheral arterial disease (PAD) and associates with high mortality after surgery. Since abnormal heart rate variability (HRV) is predictive of postoperative complications, we investigated the relations of HRV with PAD, OSA and major adverse cardiovascular and cerebrovascular events (MACCE).

**Materials and methods:**

Seventy-five patients (67±9 years) scheduled for sub-inguinal revascularization and 15 controls (63±6 years) underwent polysomnography and HRV analyses. OSA with an apnea-hypopnea index (AHI) ≥20/hour was considered significant. HRV was measured during wakefulness, S2, S3-4 and rapid eye movement (REM) sleep with time and frequency domain methods including beat-to-beat variability, low frequency (LF) and high frequency (HF) power, and detrended fluctuation analysis (DFA). MACCE was defined as cardiac death, myocardial infarction, coronary revascularization, hospitalized angina pectoris and stroke.

**Results:**

Thirty-six patients (48%) had AHI≥20/hour. During follow-up (median 52 months), 22 patients (29%) suffered a MACCE. Compared to controls, fractal correlation of HRV (scaling exponent alpha 1 measured with DFA) was weaker during S2 and evening wakefulness in all subgroups (+/-AHI≥20/hour, +/-MACCE) but only in patients with AHI≥20/hour during morning wakefulness. The LF/HF ratio was lower in all subgroups during S2 but only in patients with AHI ≥20/hour during evening or morning wake. In the covariance analysis adjusted for age, body mass index, coronary artery disease and PAD duration, the alpha 1 during morning wakefulness remained significantly lower in patients with AHI≥20/hour than in those without (1.12 vs. 1.45; p = 0.03). Decreased HF during REM (p = 0.04) and S3-4 sleep (p = 0.03) were predictive of MACCE. In analyses with all sleep stages combined, mean heart rate as well as very low frequency, LF, HF and total power were associated with OSA of mild-to-moderate severity (AHI 10-20/hour).

**Conclusions:**

HRV is altered in patients with PAD. These alterations have a limited association with OSA and MACCE.

## Introduction

Peripheral arterial disease (PAD) represents a severe form of systemic atherosclerosis with a high cardiovascular morbidity, irrespective of whether there are clinical manifestations in other vascular beds [1]. Patients with PAD undergoing vascular surgery have a poor prognosis with a 5-year mortality of approximately 30% [2]. Surgery in these patients is a high-risk procedure, mainly due to cardiac complications [2,3]. Furthermore, the underlying pathophysiological mechanisms and hence the prognostic factors are not clear. The predictive value of clinical index scores and other non-invasive methods such as echocardiography is limited, especially in patients surviving the initial month following surgery [4–6]. Consequently, evaluating the long-term prognosis in an individual patient remains a challenge to clinicians.

Heart rate variability (HRV) reflects fluctuations in autonomic nervous system activity, and it has been shown to be an important pathophysiological factor linked with cardiovascular morbidity and mortality [7,8]. Namely, HRV is perceived to reflect modulations in autonomic control of circulation with sympathetic activation, vagal compensation and renin-angiotensin system activity producing characteristic alterations [9]. Decreased or impaired HRV (measured from a 24-hour recording) has been shown to predispose to acute myocardial infarction, arrhythmias and sudden cardiac death [10,11]. However, the analysis of HRV from 24-hour recordings is time-consuming and not used in routine risk evaluation of surgical patients.

We have previously described a high prevalence of obstructive sleep apnea (OSA) in patients undergoing sub-inguinal vascular surgery [12]. We also demonstrated that OSA is significantly associated with major adverse cardiovascular and cerebrovascular events (MACCE) in these patients [13]. There is evidence suggesting that OSA can promote atherosclerosis and sympathetic activation [14]. The aim of this study was to elucidate the pathophysiological connection between OSA and the major adverse events observed earlier in these patients. To achieve this goal, we assessed autonomic dysfunction by determining the characteristics of sleep-time heart rate dynamics and whether pathological HRV is associated with OSA and MACCE in patients undergoing lower limb vascular surgery.

## Materials and methods

### Study population

This study is part of the BAROSLEEP trial (ClinicalTrials.gov identifier NCT00712946) designed to increase our understanding of the pathophysiology underpinning the poor long-term outcome of PAD patients. In this sub-study, the impact of OSA was determined on the nocturnal heart rate dynamics in the same PAD patients who have been previously shown to have highly prevalent OSA, associated with increased long-term cardiovascular morbidity and mortality [12,13]. Patients over 40 years with PAD referred to Turku University Hospital (Turku, Finland) for elective sub-inguinal revascularization were eligible to participate in this prospective study. Exclusion criteria were pre-existing OSA syndrome, clinical heart failure, atrial fibrillation, inability to co-operate, end-stage renal disease, coronary bypass within 3 years or other major surgery within 3 months prior to enrolment. In addition, 22 healthy individuals without any cardiovascular disease were recruited to serve as a control group. The participants provided written informed consent and the recruitment was carried out between April 2006 and December 2011. The study was approved by the Ethics Committee of the Hospital District of Southwest Finland, Turku, Finland (statement and approval number 404/2005).

### Data collection and interpretation

All enrolled patients underwent a detailed clinical evaluation, including preoperative echocardiography to determine left ventricular ejection fraction (LVEF). A glucose tolerance test was performed in patients without pre-existing diabetes. Coronary artery disease and hypertension were considered present if previously diagnosed. Diabetes and metabolic syndrome were diagnosed according to the established recommendations [15,16]. The ratio of blood pressures measured from the ankle and brachial artery (ankle-brachial index, ABI) was calculated as a parameter of PAD severity.

All subjects underwent preoperative overnight polysomnography (Embla/Somnologica 5.0, MedCare, Reykjavik, Iceland) as previously described [12,13]. The recordings were analyzed and sleep stages (Wake, S1, S2, S3-4, REM) were determined according to the recommended guidelines [17]. Arousals were identified using the scoring rules of the American Sleep Disorders Association [18]. Respiration was monitored with a pressure transducer attached to nasal prongs for respiratory flow. A pulse oximeter was utilized to measure arterial oxyhemoglobin saturation and plethysmography. Apnea was defined as cessation of airflow for at least 10 seconds. Hypopnea was defined as a discernible tidal volume reduction of >50%, associated with ≥4% oxyhemoglobin desaturation. OSA was diagnosed via the apnea-hypopnea index (AHI), calculated as the number of these respiratory events per hour of sleep [19]. The arterial oxyhemoglobin desaturation index was determined as the number of desaturations of at least 4% per hour of sleep and was used instead of the AHI in four patients in whom the nasal prongs had become detached during the recording. Central and obstructive apnea episodes were distinguished by evaluating the respiratory swing observed in pulse transit time, calculated from the electrocardiogram and plethysmography signals, as previously validated [20,21].

To determine nocturnal heart rate dynamics, an electrocardiogram (ECG) was recorded at the same time as the polysomnography. The interval between each individual beat was measured as the time between two normal R-peaks of the QRS complex (i.e. normal-to-normal interval, NNI). HRV was assessed using time domain, frequency domain (spectral) and non-linear (entropy and fractal) methods. The statistical time domain measurements included standard deviation of NNI, root mean square of the sum of successive differences (RMSSD) between adjacent NNI (i.e. beat-to-beat variability), proportion of NNI >50 ms (pNN50). In general, decreasing values in each time domain parameter reflect depressed HRV mainly due to compromised vagal heart rate control. Of the spectral measurements, low frequency (LF) power, high frequency (HF) power and LF/HF ratio were used to assess sympathetic activity, parasympathetic activity and sympathovagal balance, respectively, with total power reflecting overall autonomic activity [8]. The ultra-low frequency (ULF) and very low frequency (VLF) spectral components as well as normalized units of LF and HF (nLF and nHF, respectively) were also measured. Sample entropy quantifies the irregularity of an NNI time series, with increasing entropy reflecting higher complexity due to feedback regulation. The coefficient reflecting fractal correlation (scaling exponent alpha 1) of heart rate, calculated by detrended fluctuation analysis (DFA), was determined [22]. In short, a scaling exponent alpha 1 value close to (or slightly over) 1.0 reflects strong fractal correlation that is theoretically characteristic of physiological systems regulated by intricate feedback mechanisms (i.e. a healthy cardiovascular system).

A uniform length of 10 minutes of consistent sleep in the same sleep stage containing premature beats less than 10% of total number of beats was required for the HRV analysis. The ECG data from sleep periods selected for analysis were reviewed and edited manually when less frequent premature beats were detected, i.e. ectopic beats were interpolated in the middle of the adjacent normal beats. An analogous correction method has been used and detailed earlier [23]. Mean values for each HRV parameter were calculated from 1–5 different 10-minute samples of S2 sleep. HRV during rapid eye movement (REM) sleep was analyzed similarly from 1–3 averaged 10-minute epochs, and from 1–2 averaged epochs during S3-4 sleep. Wakefulness periods in the evening before sleep onset and in the morning after awakening were analyzed separately. Finally, analyses with all sleep stages (S2, S3-4, REM) combined were performed from 1–7 averaged epochs. The HRV analyses were made using WinCPRS 1.1.6.0 software for Windows (Absolute Aliens Inc., Turku, Finland). The raw HRV data (averaged per sleep stage) from all analyzed 10-minute epoch is provided in S1 Dataset. Stationarity of the NNI time series was tested with the StatAvF algorithm included in the analysis program and the method is detailed in S1 Text.

OSA was considered to be present in patients with AHI ≥5/hour, according to the guidelines of the American Academy of Sleep Medicine [19]. In the control group, subjects with mild OSA (AHI 5-15/hour) were included in the HRV analyses while those with moderate OSA (AHI 15-30/hour) or severe OSA (AHI ≥30/hour) were excluded. In the group comparisons to determine the impact of OSA on HRV, a value of AHI ≥20/hour was used as the cut-off limit for significant OSA, i.e. the same threshold that was shown to be associated with an increased risk of MACCE in our previous study [13].

In the follow-up, cardiac troponin T (cTn-T) was measured on the first 3 postoperative days. All patients were routinely examined in the outpatient ward 6 weeks and 1 year postoperatively. Subsequently, they were annually contacted by phone, and major adverse events were retrieved from hospital records. Long-term follow-up was continued until February 2013. The combined endpoint of MACCE was determined as cardiac death, acute myocardial infarction (AMI), coronary revascularization, unstable angina pectoris needing hospitalization, and stroke. AMI was determined according to the current universal definition [24].

### Statistical analysis

A pre-study power analysis was performed to determine the number of patients needed to detect statistical differences and designed in order to evaluate the preoperative predictive value of altered short-term fractal HRV (i.e. alpha 1) in different sleep stages for major postoperative adverse events in PAD patients. According to earlier studies approximately one third and up to 50% of PAD patients should develop a myocardial infarction within three postoperative days and a five-year mortality in these patients was expected to be approximately 30% [2]. Power was fixed to 85% and significance level to 0.05 in the calculations. The calculated total number of patients varied between 80 and 100, depending on the incidence of myocardial infarction between 30–50%, to reveal statistically significant differences. The alpha 1 values were expected to be lower and below 1.0 in patients suffering a MACCE (and possibly also in patients with significant OSA).

One-way analysis of variances (ANOVA) was used to test for differences in parameters. Patient groups with and without MACCE, and patients with and without significant OSA (AHI >20/hour) were compared to healthy controls using Dunnett’s post hoc procedure to correct for multiple comparisons. Non-normally distributed variables were log-transformed (natural logarithm) before any statistical analyses were performed. An analysis of covariance (ANCOVA) adjusted for age, body mass index, coronary artery disease and PAD history of less than 4 years (the latter two parameters being independently related to MACCE in our previous study) was then performed to test if there was an independent association of significant OSA and MACCE (healthy controls not included) with each HRV parameter (time and frequency domain, DFA). In the analyses with all sleep stages combined, ANOVA and ANCOVA models were also used to test for independent association of the HRV parameters with worsening OSA (AHI 10-20/hour, AHI 20-30/hour and AHI ≥30/hour with AHI <10/hour used as a control group) and MACCE. Dunnett’s post hoc adjustment for multiple comparisons was used in the ANOVA model and the ANCOVA was adjusted with age, body mass index, presence of coronary artery disease and PAD duration. Non-normally distributed variables were log-transformed (natural logarithm) in the ANOVA and ANCOVA models; the requirement for normal distribution was met after the log-transformation in these analyses. Kruskal-Wallis’s test and Mann-Whitney’s U-test with Bonferroni’s adjustment for multiple comparisons were used to test for differences in variables that were skewed after log-transformation (needed only in the descriptive comparisons between patients and controls). Exact Fisher’s test was used to test for differences in categorical variables between patient groups. P-values below 0.05 were considered statistically significant. In the statistical presentation, data is described using mean (standard deviation, SD) for normally distributed variables and median [interquartile range, IQR] for non-normally distributed variables (values before log-transformation with the width of the IQR are shown in tables for descriptive purpose). The statistical analyses were performed with SAS 9.4 software for Windows (SAS Institute Inc., Cary, North Carolina, USA).

## Results

### Patient characteristics

A total of 145 patients were eligible for the study after meeting the inclusion but without the exclusion criteria. Of these, 59 patients refused to participate, i.e. 86 patients provided their consent. Two patients had to be rejected because of insufficient sleep data due to technical problems, which meant that ultimately 84 patients were included in the final analyses, i.e. the same patients that were found to have a high prevalence of OSA associated with MACCE in our previous reports [12,13]. One study subject had type 1 diabetes mellitus (alleged latent autoimmune diabetes of adulthood), all of the other diabetic patients were type 2. Demographic and clinical characteristics measured at baseline are summarized in Table 1.

**Table 1:** Baseline characteristics according to presence of significant OSA (AHI≥20/hour) at and occurrence of MACCE during follow-up.

	Controls n = 15	AHI <20 n = 39	AHI ≥20 n = 36	No MACCE n = 53	MACCE n = 22
Age, yrs (SD)	63 (6)	65 (9)	69 (8) *	65 (9)	70 (8) †
Male, n (%)	5 (33)	24 (62)	26 (72)	35 (66)	15 (68)
BMI, kg/m^2^ (SD)	24 (2)	27 (4)	27 (4)	27 (4)	27 (3)
Metabolic syndrome, n (%)	NA	19 (49)	25 (69)	29 (55)	15 (68)
Diabetes mellitus, n (%)	NA	19 (49)	13 (36)	23 (42)	9 (43)
Hypertension, n (%)	NA	30 (77)	32 (89)	41 (77)	21 (95)
CAD, n (%)	NA	11 (28)	14 (39)	11 (21)	14 (64) ‡
Stroke, n (%)	NA	7 (18)	6 (17)	8 (15)	5 (23)
LVEF, % (SD)	70 (6)	68 (6)	59 (9) ¶	64 (8)	62 (9)
ABI ratio, (SD)	NA	0.6 (0.2)	0.6 (0.2)	0.6 (0.2)	0.6 (0.2)
PAD history, yrs [IQR]	NA	3 [4]	4 [4]	3 [6]	2 [2]
Critical ischemia, n (%)	NA	5 (13)	5 (14)	7 (13)	3 (14)

Data are mean (standard deviation = SD), median [interquartile range = IQR] or number (percentage) of patients. ABI = Ankle-brachial index, AHI = Apnea-hypopnea index, BMI = Body mass index, CAD = Coronary artery disease, LVEF = Left ventricular ejection fraction, MACCE = Major adverse cardiovascular or cerebrovascular event, OSA = Obstructive sleep apnea, PAD = Peripheral artery disease.

*: p < 0.05 vs. AHI <20/hour.

†: p < 0.05 vs. non-MACCE.

‡: p < 0.001 vs. non-MACCE

¶: p < 0.0001 vs. AHI <20/hour. One-way analysis of variance with Dunnett’s post hoc was used to test for differences in normally distributed variables between each patient group (with and without OSA/MACCE) vs. controls. Exact Fisher’s test was used to test for differences in categorical variables between patient groups (with vs. without AHI/MACCE).

ECG during NREM sleep or wakefulness was available from 75 patients. Reasons for exclusion were either excessive extrasystoles (7 patients) or unreadable ECG due to noise (2 patients). ECG during S2 sleep was available in 71 patients, and during REM sleep in 58 patients. In patients with insufficient S2, S3-4 or REM sleep, HRV was still analyzed during wakefulness. Stationarity testing of the analyzed epochs indicated that most of the data were at least moderately stationary. Overall, 6% of the included 10-minute NNI segments were non-stationary. Of the controls, a total of 6 subjects had moderate or severe OSA and were excluded along with 1 subject due to failed polysomnography. Of the included 15 controls, 3 subjects had mild OSA. The age difference between patients included in the HRV analyses and healthy controls was not statistically significant (67 vs. 63 years; p = 0.09). The patients had a significantly higher body mass index (26 vs. 24 kg/m^2^; p = 0.009) and there was a greater proportion of men (67 vs. 33%; p = 0.02) than in controls. The LVEF was significantly lower in patients than in controls (64% vs. 70%; p = 0.01). There were no significant differences in the use of medications, including beta-blockers between patients with and without AHI ≥20/hour or MACCE (Table 2).

**Table 2:** Long-term medications of patients according to significant OSA (AHI≥20/hour) at baseline and occurrence of MACCE during follow-up.

	AHI <20 n = 39	AHI ≥20 n = 36	No MACCE n = 53	MACCE n = 22
Any antihypertensive Beta-blocker ACE-inhibitor or ATR-blocker 3 or more antihypertensives	33 (85) 21 (54) 23 (59) 8 (21)	34 (94) 28 (78) 21 (58) 10 (28)	45 (85) 32 (60) 30 (57) 12 (23)	22 (100) 17 (77) 14 (64) 6 (27)
Statins	24 (62)	22 (61)	33 (62)	13 (59)
Any antithrombotic ASA Clopidogrel Warfarin	37 (95) 34 (87) 11 (28) 4 (10)	32 (89) 31 (86) 7 (19) 2 (6)	48 (91) 45 (85) 12 (23) 4 (8)	21 (96) 20 (91) 6 (27) 2 (9)
Opiates	7 (18)	12 (33)	15 (28)	4 (18)
Benzodiazepines	5 (13)	5 (14)	9 (17)	1 (5)

Data are number (percentage) of patients. ACE = Angiotensin converting enzyme, AHI = Apnea-hypopnea index, ASA = Acetyl salicylic acid, ATR = Angiotensin receptor, OSA = Obstructive sleep apnea, MACCE = Major adverse cardiovascular or cerebrovascular event. Exact Fisher’s test was used to test for differences between patient groups (with vs. without AHI/MACCE); p = not significant for all.

Of the 75 patients that were included in the HRV analyses, 36 (48%) had significant OSA (AHI ≥20/hour). Not surprisingly, the arousal index in patients with AHI ≥20/hour was significantly higher than in those without (25/hour vs. 17/hour; p = 0.001) but was not different between patients with and without MACCE (22/hour vs. 21/hour; p = 0.7). The arousal index in the healthy controls did not significantly differ from that of the patients with AHI <20/hour (13/hour vs. 17/hour; p = 0.1). The average proportion of central apnea episodes was 24% in the whole study population. There was no significant difference in the proportion of central apneas between patients with and without AHI ≥20/hour (23% vs. 25%; p = 0.2), or with and without MACCE (23% vs. 24%; p = 0.7).

### Events during follow-up

During follow-up (median 52 months), a total of 22 patients (29%) suffered a MACCE and 17 patients had died (all-cause mortality 23%). There were seven cardiac deaths (as the first major event), nine AMIs, four episodes of unstable angina pectoris requiring hospitalization or coronary revascularization and two strokes (one ischemic, one hemorrhagic). There was a significant difference in the occurrence of MACCE between patients with and without AHI ≥20/hour (16 vs. 6 events, p = 0.01). Two of the eleven patients in whom HRV could not be analyzed, suffered a fatal MACCE during the follow-up (two with unanalyzable ECG, one with failed polysomnography).

### HRV differences in patients vs. controls

Comparisons between patient groups (with and without AHI ≥20/hour, with and without MACCE) and controls are detailed in TAbles 3, 4, 5, 6 and 7 (S2 sleep, REM sleep, evening and morning wakefulness, S3-4 sleep, respectively). In general, time domain measures of HRV showed few differences when patient groups (with and without AHI ≥20/hour, with and without MACCE) were compared to controls. These included lower standard deviation of NNI in patients with AHI ≥20/hour and those with MACCE during morning wakefulness and lower mean NNI in patients with MACCE during REM sleep. In the frequency domain analyses, LF power was consistently lower in PAD patients than in controls but HF was generally higher when measured in normalized units. This resulted in significantly lower LF/HF ratio in all patient groups vs. controls, which was most clearly seen during S2 and REM sleep. ULF power and VLF power were significantly lower in patients AHI ≥20/hour and those with MACCE during morning wakefulness. In the non-linear analyses, sample entropy was almost identical across all patient groups and controls during both sleep and wakefulness. In the DFA analyses, the scaling exponent alpha 1 was consistently lower in all groups of PAD patients vs. controls. This trend was most significant during S2 sleep and evening wakefulness but mean alpha 1 values remained close to 1.0 or above in all analyses except S3-4 sleep. During S3-4 sleep, HRV parameters in any of the patient groups did not differ from those of the controls.

**Table 3:** HRV characteristics in S2 sleep according to OSA severity and MACCE.

	Controls n = 15	AHI <20 n = 39	AHI ≥20 n = 32	No MACCE n = 51	MACCE n = 20
HR, 1/min	57 (52–65)	61 (54–71)	61 (52–72*)	61 (53–71)	63 (54–72)
NNI Min, ms	925 (80)	847 (149)	833 (113) *	844 (135)	830 (131)
NNI Max, ms	1164 (115)	1116 (201)	1146 (239)	1139 (205)	1104 (253)
NNI Mean, ms	1047 (95)	977 (173)	980 (141)	987 (154)	956 (171)
NNI Dev, ms	37 [18]	40 [28]	34 [39]	40 [26]	27 [35]
NNI RMSSD, ms	23 [25]	29 [23]	26 [37]	29 [24]	26 [25]
NNI pNN50, %	2.2 [19.0]	5.2 [12.0]	5.2 [14.6]	6.2 [17.6]	3.9 [10.4]
NNI SampEn	1.5 (0.3)	1.5 (0.3)	1.4 (0.3)	1.5 (0.3)	1.4 (0.3)
NNIS Total, ms^2^	1209 [816]	1306 [1789]	981 [2988]	1306 [2657]	697 [2563]
NNIS ULF, ms^2^	29 [38]	23 [48]	40 [54]	33 [42]	39 [97]
NNIS VLF, ms^2^	305 [354]	471 [1082]	445 [1357]	471 [1049]	368 [1236]
NNIS LF, ms^2^	357 [672]	293 [741]	188 [645]	387 [779]	92 [414]
NNIS HF, ms^2^	100 [297]	158 [303]	140 [473]	165 [462]	87 [209]
NNIS LF/HF	4.8 [7.7]	2.5 [3.3] *	1.9 [3.1] †	2.4 [3.1] *	1.5 [3.3] *
NNIS nLF, nu	75 (19)	60 (23) *	58 (21) *	60 (21) *	57 (24) *
NNIS nHF, nu	23 (17)	38 (21) *	37 (17) *	37 (19) *	38 (20) *
NNI Alpha 1	1.29 (0.33)	1.05 (0.33) *	1.00 (0.34) *	1.04 (0.31) *	1.00 (0.38) *

Data are mean (standard deviation) or median [interquartile range] except for mean (range) for heart rate (HR). AHI = Apnea-hypopnea index, Alpha 1 = Fractal scaling exponent alpha 1, Dev = Standard deviation of NNI, HF = Power in the high frequency range (0.15–0.4 Hz), LF = Power in low frequency range 0.04–0.15 Hz), MACCE = Major adverse cardiovascular and cerebrovascular event, ms = millisecond, nHF = normalized HF ratio, nLF = normalized LF ratio, NNI = normal-to-normal interval (i.e. time between normal beats in the electrocardiogram), NNIS = NNI spectrum, pNN50 = Proportion of NNI >50 ms, RMSSD = Root mean square of the sum of successive differences (between adjacent normal-to-normal intervals, i.e. beat-to-beat variability), SampEn = Sample entropy, Total = Total power of the NNI spectrum (i.e. overall autonomic activity), ULF = Power in the ultra-low frequency range (≤0.003 Hz), VLF = Power in the very low frequency range (0.003–0.04 Hz).

*: p < 0.05 vs. controls

†: p < 0.01 vs. controls. One-way analysis of variance with Dunnett’s post hoc was used to test for differences between each patient group (with and without OSA/MACCE) vs. controls. Analysis of covariance was used to test for independent differences between patient groups (with vs. without OSA/MACCE). Non-normally distributed variables were log-transformed (natural logarithm) before the statistical analyses (non-transformed values with IQRs in square brackets shown in tables).

**Table 4:** HRV characteristics in evening wake according to OSA severity and MACCE.

	Controls n = 15	AHI <20 n = 36	AHI ≥20 n = 32	No MACCE n = 49	MACCE n = 19
HR, 1/min	63 (55–77)	67 (58–80)	66 (58–78)	67 (58–78)	67 (58–82)
NNI Min, ms	778 (111)	752 (117)	772 (105)	771 (105)	736 (125)
NNI Max, ms	1095 (173)	1028 (169)	1041 (169)	1032 (165)	1038 (178)
NNI Mean, ms	956 (158)	899 (129)	905 (132)	902 (131)	901 (127)
NNI Dev, ms	44 [18]	34 [30]	34 [19]	33 [21]	36 [31]
NNI RMSSD, ms	23 [14]	22 [15]	22 [18]	21 [13]	24 [32]
NNI pNN50, %	2.8 [4.5]	1.5 [3.8]	3.0 [5.8]	1.5 [4.4]	2.5 [7.5]
NNI SampEn	1.2 (0.2)	1.3 (0.3)	1.3 (0.3)	1.3 (0.3)	1.2 (0.3)
NNIS Total, ms^2^	1541 [1159]	803 [2148]	1067 [1129]	910 [1420]	1012 [2561]
NNIS ULF, ms^2^	179 [155]	54 [287]	62 [145]	53 [188]	85 [255]
NNIS VLF, ms^2^	1108 [1000]	414 [98]	438 [710]	424 [825]	427 [816]
NNIS LF, ms^2^	463 [518]	247 [341]	186 [299] *	227 [328]	180 [651]
NNIS HF, ms^2^	104 [128]	63 [154]	84 [145]	72 [128]	91 [356]
NNIS LF/HF	5.3 [6.3]	3.2 [4.3]	2.0 [3.6] *	2.6 [3.4]	2.2 [3.9]
NNIS nLF, nu	81 (10)	70 (18)	63 (20) †	69 (17)	60 (24) †
NNIS nHF, nu	18 (9)	27 (15)	33 (18) †	28 (15)	35 (21) †
NNI Alpha 1	1.37 (0.21)	1.16 (0.32) *	1.11 (0.33) *	1.15 (0.30) *	1.08 (0.39) *

Data are mean (standard deviation) or median [interquartile range] except for mean (range) for heart rate (HR). AHI = Apnea-hypopnea index, Alpha 1 = Fractal scaling exponent alpha 1, Dev = Standard deviation of NNI, HF = Power in the high frequency range (0.15–0.4 Hz), LF = Power in low frequency range 0.04–0.15 Hz), MACCE = Major adverse cardiovascular and cerebrovascular event, ms = millisecond, nHF = normalized HF ratio, nLF = normalized LF ratio, NNI = normal-to-normal interval (i.e. time between normal beats in the electrocardiogram), NNIS = NNI spectrum, pNN50 = Proportion of NNI >50 ms, RMSSD = Root mean square of the sum of successive differences (between adjacent normal-to-normal intervals, i.e. beat-to-beat variability), SampEn = Sample entropy, Total = Total power of the NNI spectrum (i.e. overall autonomic activity), ULF = Power in the ultra-low frequency range (≤0.003 Hz), VLF = Power in the very low frequency range (0.003–0.04 Hz).

*: p <0.05 vs. controls

†: p <0.01 vs. controls. One-way analysis of variance with Dunnett’s post hoc was used to test for differences between each patient group (with and without OSA/MACCE) vs. controls. Analysis of covariance was used to test for independent differences between patient groups (with vs. without OSA/MACCE). Non-normally distributed variables were log-transformed (natural logarithm) before the statistical analyses (non-transformed values with IQRs in square brackets shown in tables).

**Table 5:** HRV characteristics in morning wake according to OSA severity and MACCE.

	Controls n = 15	AHI <20 n = 30	AHI ≥20 n = 30	No MACCE n = 43	MACCE n = 17
HR, 1/min	64 (54–87)	68 (59–83)	68 (57–80)	68 (59–83)	67 (56–79)
NNI Min, ms	688 (129)	727 (121)	747 (102)	727 (118)	764 (92)
NNI Max, ms	1121 (119)	1024 (212)	1055 (205)	1025 (190)	1076 (248)
NNI Mean, ms	933 (102)	878 (158)	887 (120)	878 (145)	898 (126)
NNI Dev, ms	52 [46]	46 [36]	37 [27] *	43 [34]	34 [31] *
NNI RMSSD, ms	20 [16]	20 [17]	20 [21]	20 [18]	18 [16]
NNI pNN50, %	2.1 [4.4]	1.6 [5.4]	2.2 [5.8]	1.8 [6.1]	2.2 [4.9]
NNI SampEn	1.0 (0.3)	1.1 (0.3)	1.1 (0.3)	1.1 (0.3)	1.1 (0.3)
NNIS Total, ms^2^	2174 [4952]	1430 [3487]	996 [2043] *	1193 [2926]	943 [1903]
NNIS ULF, ms^2^	223 [881]	140 [488]	69 [225] *	118 [455]	103 [322] *
NNIS VLF, ms^2^	1482 [2674]	762 [2309]	580 [1096] *	633 [1692]	490 [1096] *
NNIS LF, ms^2^	395 [1038]	254 [283]	218 [371]	254 [323]	173 [367]
NNIS HF, ms^2^	75 [120]	76 [99]	105 [110]	91 [110]	61 [116]
NNIS LF/HF	5.4 [5.0]	3.9 [3.3]	2.5 [3.5] * ‡	3.7 [3.7]	3.0 [3.7]
NNIS nLF, nu	80 (14)	73 (19)	64 (20) *	69 (20)	67 (20)
NNIS nHF, nu	20 (12)	24 (18)	32 (16) *	28 (17)	29 (18)
NNI Alpha 1	1.5 [0.18]	1.34 (0.35)	1.12 (0.34)†‡	1.25 (0.36)	1.20 (0.35)

Data are mean (standard deviation) or median [interquartile range] except for mean (range) for heart rate (HR). AHI = Apnea-hypopnea index, Alpha 1 = Fractal scaling exponent alpha 1, Dev = Standard deviation of NNI, HF = Power in the high frequency range (0.15–0.4 Hz), LF = Power in low frequency range 0.04–0.15 Hz), MACCE = Major adverse cardiovascular and cerebrovascular event, ms = millisecond, nHF = normalized HF ratio, nLF = normalized LF ratio, NNI = normal-to-normal interval (i.e. time between normal beats in the electrocardiogram), NNIS = NNI spectrum, pNN50 = Proportion of NNI >50 ms, RMSSD = Root mean square of the sum of successive differences (between adjacent normal-to-normal intervals, i.e. beat-to-beat variability), SampEn = Sample entropy, Total = Total power of the NNI spectrum (i.e. overall autonomic activity), ULF = Power in the ultra-low frequency range (≤0.003 Hz), VLF = Power in the very low frequency range (0.003–0.04 Hz).

*: p <0.05 vs. controls

†: p <0.01 vs. controls

‡: p<0.05 vs. AHI <20/hour (prior to adjustment for age, body mass index, coronary artery disease and PAD history). One-way analysis of variance with Dunnett’s post hoc was used to test for differences between each patient group (with and without OSA/MACCE) vs. controls. Analysis of covariance was used to test for independent differences between patient groups (with vs. without OSA/MACCE). Non-normally distributed variables were log-transformed (natural logarithm) before the statistical analyses (non-transformed values with IQRs in square brackets shown in tables). Kruskal-Wallis’s test and Mann-Whitney’s U-test with Bonferroni’s adjustment for multiple comparisons were used to test for differences in variables that were skewed after log-transformation.

**Table 6:** HRV characteristics in REM sleep according to OSA severity and MACCE.

	Controls n = 14	*AHI <20* *n = 33*	AHI ≥20 n = 25	No MACCE n = 46	MACCE n = 12
HR, 1/min	58 (55–71)	*63 (53–76)*	64 (52–77)	63 (52–75)	67* (59‡-80)
NNI Min, ms	850 (100)	*788 (117)*	783 (119)	795 (118)	752 (112)
NNI Max, ms	1189 (145)	*1122 (203)*	1145 (234)	1161 (217)	1018 (173) ‡
NNI Mean, ms	1031 (117)	*950 (143)*	939 (143)	960 (140)	890 (140) *
NNI Dev, ms	42 [25]	*42 [31]*	44 [33]	45 [38]	36 [30]
NNI RMSSD, ms	21 [14]	*23 [15]*	28 [25]	26 [25]	18 [23]
NNI pNN50, %	1.5 [5.7]	*1*.*8 [8*.*8]*	2.7 [4.6]	2.7 [9.3]	1.4 [4.1] ‡
NNI SampEn	1.1 (0.3)	*1*.*2 (0*.*3)*	1.1 (0.3)	1.2 (0.3)	1.0 (0.3)
NNIS Total, ms^2^	1522 [2381]	*1605 [2726]*	1714 [2842]	1863 [3624]	967 [1557]
NNIS ULF, ms^2^	126 [307]	*199 [300]*	128 [332]	195 [312]	86 [121]
NNIS VLF, ms^2^	933 [953]	*1001 [1838]*	1049 [1692]	1172 [1838]	525 [1144]
NNIS LF, ms^2^	417 [1080]	*295 [671]*	224 [461]	276 [671]	192 [225] *
NNIS HF, ms^2^	53 [140]	*85 [138]*	94 [176]	96 [205]	35 [86] ‡
NNIS LF/HF	10.3 [14.7]	*3*.*9 [4*.*2]* *	1.8 [3.0] †	2.5 [3.3] †	3.6 [3.9]
NNIS nLF, nu	82 (17)	*70 (18)*	62 (18) †	66 (17) †	68 (22)
NNIS nHF, nu	17 (16)	*27 (16)*	35 (17) †	31 (16) †	27 (19)
NNI Alpha 1	1.64 [0.46]	*1*.*18 (0*.*34)*	1.06 (0.37) *	1.10 (0.32) *	1.21 (0.48)

Data are mean (standard deviation) or median [interquartile range] except for mean (range) for heart rate (HR). AHI = Apnea-hypopnea index, Alpha 1 = Fractal scaling exponent alpha 1, Dev = Standard deviation of NNI, HF = Power in the high frequency range (0.15–0.4 Hz), LF = Power in low frequency range 0.04–0.15 Hz), MACCE = Major adverse cardiovascular and cerebrovascular event, ms = millisecond, nHF = normalized HF ratio, nLF = normalized LF ratio, NNI = normal-to-normal interval (i.e. time between normal beats in the electrocardiogram), NNIS = NNI spectrum, pNN50 = Proportion of NNI >50 ms, RMSSD = Root mean square of the sum of successive differences (between adjacent normal-to-normal intervals, i.e. beat-to-beat variability), SampEn = Sample entropy, Total = Total power of the NNI spectrum (i.e. overall autonomic activity), ULF = Power in the ultra-low frequency range (≤0.003 Hz), VLF = Power in the very low frequency range (0.003–0.04 Hz).

*: p < 0.05 vs. controls

†: p < 0.01 vs. controls

‡: p<0.05 between patients with and without MACCE (prior to adjustment for age, body mass index, coronary artery disease and PAD history). One-way analysis of variance with Dunnett’s post hoc was used to test for differences between each patient group (with and without OSA/MACCE) vs. controls. Analysis of covariance was used to test for independent differences between patient groups (with vs. without OSA/MACCE). Non-normally distributed variables were log-transformed (natural logarithm) before the statistical analyses (non-transformed values with IQRs in square brackets shown in tables). Kruskal-Wallis’s test and Mann-Whitney’s U-test with Bonferroni’s adjustment for multiple comparisons were used to test for differences in variables that were skewed after log-transformation.

**Table 7:** HRV characteristics in S3-4 sleep according to OSA severity and MACCE.

	Controls n = 7	AHI <20 n = 18	AHI ≥20 n = 13	No MACCE n = 22	MACCE n = 9
HR, 1/min	58 (54–64)	62 (58–69)	59 (53–66)	61 (54–67)	63 (58–69)
NNI Min, ms	939 (81)	874 (156)	910 (126)	897 (149)	870 (132)
NNI Max, ms	1113 (84)	1042 (173)	1138 (174)	1103 (187)	1031 (148)
NNI Mean, ms	1028 (80)	963 (155)	1020 (127)	999 (147)	956 (142)
NNI Dev, ms	24 [12]	23 [19]	25 [18]	26 [18]	19 [14]
NNI RMSSD, ms	21 [17]	23 [15]	23 [20]	25 [17]	13 [13] †
NNI pNN50, %	0.8 [9.0]	1.7 [7.8]	1.4 [12.9]	6.0 [16.5]	0.8 [1.2]
NNI SampEn	1.7 (0.4)	1.6 (0.3)	1.7 (0.1)	1.7 (0.2)	1.6 (0.3)
NNIS Total, ms^2^	454 [377]	380 [965]	648 [998]	461 [940]	292 [544]
NNIS ULF, ms^2^	20 [26]	8 [17]	17 [27]	13 [22]	15 [20]
NNIS VLF, ms^2^	181 [198]	112 [126]	186 [416]	148 [138]	167 [231]
NNIS LF, ms^2^	229 [219]	69 [292]	134 [504]	115 [487]	58 [214]
NNIS HF, ms^2^	89 [241]	112 [148]	153 [234]	162 [216]	25 [134] †
NNIS LF/HF	1.5 [5.4]	0.95 [2.5]	1.3 [1.4]	0.83 [0.87]	2.3 [1.9] *
NNIS nLF, nu	63 (22)	49 (25)	55 (16)	46 (21)	65 (15) *
NNIS nHF, nu	35 (21)	48 (24)	44 (16)	52 (21)	33 (14) *
NNI Alpha 1	1.07 [0.66]	0.93 (0.36)	0.95 (0.22)	0.88 (0.31)	1.10 (0.26)

Data are mean (standard deviation) or median [interquartile range] except for mean (range) for heart rate (HR). AHI = Apnea-hypopnea index, Alpha 1 = Fractal scaling exponent alpha 1, Dev = Standard deviation of NNI, HF = Power in the high frequency range (0.15–0.4 Hz), LF = Power in low frequency range 0.04–0.15 Hz), MACCE = Major adverse cardiovascular and cerebrovascular event, ms = millisecond, nHF = normalized HF ratio, nLF = normalized LF ratio, NNI = normal-to-normal interval (i.e. time between normal beats in the electrocardiogram), NNIS = NNI spectrum, pNN50 = Proportion of NNI >50 ms, RMSSD = Root mean square of the sum of successive differences (between adjacent normal-to-normal intervals, i.e. beat-to-beat variability), SampEn = Sample entropy, Total = Total power of the NNI spectrum (i.e. overall autonomic activity), ULF = Power in the ultra-low frequency range (≤0.003 Hz), VLF = Power in the very low frequency range (0.003–0.04 Hz).

*: p <0.05 between patients with and without MACCE

†: p<0.01 between patients with and without MACCE (prior to adjustment for age, body mass index, coronary artery disease and PAD history). One-way analysis of variance with Dunnett’s post hoc was used to test for differences between each patient group (with and without OSA/MACCE) vs. controls. Analysis of covariance was used to test for independent differences between patient groups (with vs. without OSA/MACCE). Non-normally distributed variables were log-transformed (natural logarithm) before the statistical analyses (non-transformed values with IQRs in square brackets shown in tables).

### Association of HRV with OSA and MACCE

The covariance analyses showed that HRV during S2 sleep, as well as either evening or morning wakefulness, displayed no association with MACCE. Furthermore, none of the HRV parameters during S2 sleep or evening wakefulness associated significantly with OSA. During morning wakefulness, patients with significant OSA (AHI ≥20/hour) had significantly lower scaling exponent alpha 1 than those without such severe OSA (1.12 vs. 1.45; p = 0.02) and this difference remained after adjusting for age, body mass index, coronary artery disease and PAD history (p = 0.03). The unadjusted LF/HF ratio was also significantly lower in patients with AHI ≥20/hour than in patients with AHI ≤20/hour (2.48 vs. 3.93; p = 0.046) during morning wakefulness but this difference disappeared after the adjustments. No HRV parameter was predictive of significant OSA during REM or S3-4 sleep. Patients with MACCE had lower maximum NNI (1018 ms vs. 1161 ms; p = 0.04), pNN50 (1.4% vs. 2.7%; p = 0.04) and HF power (35 ms^2^ vs. 96 ms^2^; p = 0.01) during REM sleep than those without, and the difference in HF power remained after the adjustments (p = 0.04). Patients suffering a MACCE had lower RMSSD (13 ms vs. 25 ms; p = 0.009), HF power (25 ms^2^ vs. 162 ms^2^; p = 0.004) and normalized HF units (33 nu vs. 52 nu; p = 0.02) as well as higher LF/HF ratio (2.3 vs. 0.83; p = 0.04) and normalized LF units (65 nu vs. 46 nu; p = 0.02) during S3-4 sleep. Of these, the differences in RMSSD (p = 0.04), HF power (p = 0.03) and normalized HF units (p = 0.048) remained significant after the adjustments.

In the analyses with all sleep stages combined, lower LF power (114 ms^2^ vs. 312 ms^2^; p = 0.04) and lower HF power (68 ms^2^ vs. 150 ms^2^; p = 0.03) were associated with MACCE but these differences were not significant after adjusting for age, body mass index, coronary artery disease and PAD history. The presence of clinical coronary artery disease had the greatest effect on statistical significance in the adjustments. When patients with worsening OSA were compared to those with AHI <10/hour, higher minimum (891 ms vs. 767 ms; p = 0.008), maximum (1198 ms vs. 1011; p = 0.02) and mean (1040 vs. 884; p = 0.006) NNI as well as standard deviation (47 ms vs. 25 ms; p = 0.03) of NNI of the statistical time domain parameters were associated with AHI 10-20/hour. Of the spectral parameters, higher VLF (1435 ms^2^ vs. 312 ms^2^; p = 0.02), LF (465 ms^2^ vs. 153 ms^2^; p = 0.04), HF (249 ms^2^ vs. 69 ms^2^; p = 0.02) and total (2080 ms^2^ vs. 615 ms^2^; p = 0.02) power were predictive of AHI 10-20/hour. Patients with AHI ≥30/hour had higher standard deviation (38 ms vs. 25 ms; p = 0.048) of NNI and higher RMSSD (31 ms vs. 21 ms; p = 0.02) as well as higher HF (164 ms^2^ vs. 69 ms^2^; p = 0.02) power than those with AHI <10/hour. These differences were observed after adjusting for age, body mass index, coronary artery disease and PAD history but none of the parameters was predictive of AHI 20-30/hour. VLF power also was higher in patients with AHI 20-30/hour and ≥30/hour but the increase was less pronounced and not statistically significant. The HRV parameters in the analyses with combined sleep stages are detailed in Table 8 (S1 Table).

## Discussion

As far as we are aware, this is the first study to assess HRV in a patient population with PAD and OSA. It revealed that the characteristics of sleep-time heart rate dynamics in patients with systemic atherosclerosis differ from those of healthy controls, implying that the autonomic regulation of heart rate during sleep is altered in these patients. However, the alterations in heart rate dynamics having an independent association with MACCE were observed in a small number of parameters and there was a non-linear association with the severity of OSA. These findings provide insights into risk stratification of patients undergoing sub-inguinal vascular surgery due to lower limb atherosclerosis.

There is convincing evidence that decreased HRV (measured from 24-hour recordings) is associated with cardiac death or myocardial infarction following major surgery [25,26]. Several studies suggest HRV to be a more powerful prognostic factor of mortality than established clinical parameters such as reduced LVEF in patients with cardiovascular disease and that impaired HRV is independently associated with one-year mortality after non-cardiac surgery [8,11,25,27]. Some studies have shown nonlinear measurements of HRV such as DFA (from 24-hour recordings or periods of 3–5 hours) to be superior to time and frequency domain methods [27,28]. Impaired nocturnal HRV (periods of 3–5 hours) has also been shown to promote postoperative myocardial ischemia, emphasizing the cardiovascular risk associated with sleep [28].

Although it is evident that decreased HRV is clearly detrimental in ischemic heart disease, the association between HRV and PAD is more complex. In contrast to our present findings, some earlier studies have shown that decreased HRV (measured from 24-hour recordings) in patients with PAD is independently associated with adverse cardiac events and thus is superior to clinical risk scoring [26,29]. In the present study, we observed decreasing LF power in patients with PAD compared to controls while the HF power increased to a lesser extent. One earlier study detected an *increased* LF/HF ratio in patients with PAD (measured from a 30-minute recording), whereas another study observed a trend towards a *decreased* LF/HF ratio and a favorable effect of exercise training on HRV (5-minute recording) [30,31]. Despite demonstrating altered heart rate dynamics in patients with surgical PAD, the link of these alterations with MACCE was very limited. This may be due to the fact that PAD patients undergoing revascularization represent one of the most high-risk surgical populations. Therefore, HRV is likely to be adversely disturbed in the majority of these patients, limiting the prognostic viability of HRV measurements.

The LF/HF ratio has been perceived to reflect the sympathovagal balance, at least to some extent (i.e. sympathetic activation increases the ratio) as LF and HF are mainly affected by sympathetic and vagal activity, respectively. When the LF/HF ratio increases under physiological conditions (head-up tilt test and low-intensity exercise, measured from short-term recordings), alpha 1 increases, indicative of an enhanced fractal correlation of heart rate [32]. In an earlier study (based on cold hand and cold face tests with 2-minute ECG recordings), this self-similarity seems to be disrupted by persistent sympathetic activation, specifically when both the sympathetic and vagal branches (i.e. accentuated sympathovagal interaction) of the autonomic nervous system are activated, resulting in compromised cardiovascular adaptability [33]. This kind of autonomic dysfunction (HRV measured from 24-hour recordings and periods of 7–9 hours during day and night) is known to occur in heart failure and has been shown to be associated with increased mortality [34]. One could postulate that OSA with PAD could represent a similar pathophysiological condition. Increased sympathetic and decreased parasympathetic tone reflected by increased LF/HF ratio during both REM and non-REM sleep has been shown in patients with OSA in an earlier study [35]. However, the nature of sleep-disordered breathing in this study is somewhat controversial due to the remarkably high proportion of central apneas. Therefore, the actual effect of the frequent apneas (both obstructive and central), arousals and intermittent hypoxia on the LF/HF ratio and the meaning of the results is undetermined. Since much of the HF power is related to sinus arrhythmia caused by breathing, this spectral component may be particularly affected by the highly prevalent sleep-disordered breathing in our patients. Indeed, the use of the LF/HF ratio as a measure of cardiac sympathovagal balance has been questioned and must be interpreted with caution in general [36]. Taken together, it seems that the use of the LF/HF ratio is not feasible in patients with OSA and severe PAD.

OSA promotes sympathetic activation [14,37]. Corresponding alterations in spectral HRV analysis (from 2-minute and 10-minute periods) have been shown previously in patients with OSA both during sleep and wakefulness [38,39]. One previous study showed the scaling exponent alpha 1 (measured from 5-minute segments) correlating closely with the AHI but this was not confirmed in another study (HRV measured from approximately 6-hour recordings) [40,41]. In the present study, the minor alterations observed in heart rate dynamics in PAD patients were not independently associated with MACCE except for few selected parameters in the relatively small number of patients in which REM or S3-4 sleep was available for analysis. Furthermore, these alterations did not clearly indicate pathological sympathovagal balance since there was no significant deterioration of fractal heart rate dynamics related to OSA, i.e. the average scaling exponent values remained close to 1 or above (but less so during sleep) despite being significantly lower in patients with AHI ≥20/hour during morning wakefulness than in those without such severe OSA. This finding is also easily explained by the potential disruption of neurocirculatory regulation caused by the frequent apneas and disturbed sleep. An earlier study showed that alpha 1 less than 1.0 was predictive of increased cardiac mortality in a general elderly population [42]. Thus, DFA measurements obtained from an individual patient provide limited information with respect to determining prognosis and guiding clinical decisions in the patients of this study. Taken together, although the alterations in heart rate dynamics in patients with severe PAD may to some extent be related to OSA, the increased risk conferred by OSA in this patient group is not explained by cardiac autonomic dysfunction alone. In addition, the prognostic viability of the observed differences that were associated with MACCE is further limited by the difficulty to obtain data from REM or S3-4 sleep for analysis.

As discussed above, impaired HRV has been shown to be predictive of increased morbidity and mortality in several patient groups and even in smaller populations than in this study [24,29]. Despite these previous findings, we were unable to demonstrate widespread pathologic alterations in heart rate dynamics predictive of MACCE in patients with PAD undergoing vascular surgery. Since the trend towards decreasing alpha 1 during S2 sleep and wakefulness in patients suffering a MACCE was quite weak, it does not seem likely that clinically significant deterioration of fractal heart rate control would have become evident in a larger sample. However, alpha 1 was lower in all patient subgroups than in controls, suggesting that autonomic control of HRV is altered in patients with PAD. As for the observed differences in the spectral measures of HRV, especially LF power was decreased consistently in all patient groups. The decreased HF power during REM and S3-4 sleep was an expectable finding and reflects impaired vagal compensation in patients suffering a MACCE. As stated above, all of our patients had a severe form of PAD (requiring surgery). In our previous study 85% of patients with surgical PAD have at least mild OSA (AHI ≥5/hour) with a significant amount of concomitant central apnea [12]. The high prevalence of OSA has later been demonstrated in patients with less severe PAD as well [43,44]. Therefore, it is feasible to suggest that their autonomic nervous system function, with or without OSA is altered in such a way that potential differences are obscured. Conversely, it is also possible that due to our selection criteria (e.g. excluding patients with heart failure) and concomitant conservative treatment, the patients examined in this study had retained their autonomic adaptability to a certain degree. To complicate matters further, the alterations caused by PAD and OSA with a prominent central component may interact in such a way that limits the clinical relevance of the HRV parameters used in this study, and accounting for the severity of OSA may not be essential when HRV is analyzed in surgical PAD patients.

When HRV was analyzed without specific sleep stage selection, independent alterations predictive for MACCE (after adjustments) were not found. With regards to OSA, more differences were observed. Patients with AHI 10-20/hour had remarkably higher power in most of the spectral parameters especially in the VLF band. However, the findings did not correlate with the worsening of OSA since the alterations in HRV were different or not significant as the AHI progressed. For example, a further increase in VLF power should have been expected [45]. In contrast with this earlier study, LF and HF power or their normalized units did not correlate consistently with increase in the AHI. We suggest that this may result from the different effects of OSA, central apnea and PAD. These results show that a dichotomic cut-off limit for the AHI cannot be used to assess the effect of OSA on HRV. It also seems that calculating nocturnal HRV generally during sleep is clinically feasible, rather than from selected sleep stages. Especially in OSA, the detrimental effects on autonomic function are not limited to the actual time of the apnea but tend to persist over a longer period.

There are important limitations to be considered. The incidence of MACCE and mortality were lower than in earlier studies that have assessed predictors of adverse outcome following vascular surgery [2]. Furthermore, HRV could not be analyzed in eleven patients, two of whom suffered a fatal MACCE during follow-up. These factors, along with the relatively small number of patients and controls may still have obscured differences that would have become evident in a larger sample. Nonetheless, we have previously observed the association of OSA with MACCE in the same population and this relationship was also seen in the smaller number of patients that underwent HRV analyses. Therefore, the absence of significant prognostic factors in heart rate dynamics is not explained by sample size alone. However, the possibility still remains that existing HRV alterations associated with MACCE simply did not become evident in this particular group of patients (type II statistical error). Furthermore, dividing the study population into four subgroups simultaneously accounting for significant OSA and MACCE (i.e. AHI<20/hour-MACCE, AHI<20/hour+MACCE, AHI≥20/hour-MACCE, AHI≥20/hour+MACCE) would have been optimal. With the size of this study sample, this would have resulted in too small subgroups and not being able to do so is a weakness of this study. While we were able to analyze HRV with 10/hour increments in the AHI, the small size of the subgroups may have obscured potential effects of worsening OSA. Non-stationarities in an HRV analysis are known to bias results toward sympathetic predominance and potentially obscure differences between groups [46]. Ensuring stationarity in any real-life biosignal is very challenging. Our results were obtained from relatively stationary data but existing non-stationarities may still have reduced the power of this study to predict adverse outcome. Finally, our approach of selecting specific sleep stages for analysis makes comparison to earlier studies challenging. Although the findings of our study support the current practice of not including HRV analysis in clinical protocols, more studies will be needed in larger, more heterogenous, populations (i.e. more variety in the severity of PAD and co-morbidities) to elucidate the significance of the HRV alterations that were detected in this study.

In conclusion, HRV was altered in all PAD patients in general but these differences offered limited explanation to the previously shown association of OSA with MACCE in patients with PAD undergoing sub-inguinal vascular surgery. Nocturnal HRV without specific sleep stage selection was affected by the AHI mainly in the frequency domain but these alterations did not directly correlate to worsening of OSA in this study. Differences in HRV associated with MACCE during sleep were still observed, showing that PAD is associated with neurocirculatory dysregulation that increases morbidity and mortality. Further studies are needed to identify potential PAD patient groups in which preoperative risk stratification with HRV analysis could be helpful.

## Supporting information

S1 Dataset HRV data contains the raw HRV data from each individual study subject. (XLS)

S1 Text Stationarity analysis contains the detailed description of the StatAvF algorithm. (DOC)

S1 Table Table 8 contains tabular data of HRV parameters with all sleep stages combined referred in the results section as Table 8. (DOC)
