# Supplementary material for: Alterations in heart rate variability in patients with peripheral arterial disease requiring surgical revascularization have limited association with postoperative major adverse cardiovascular and cerebrovascular events
Source: PLoS One. 2018 Sep 13;13(9):e0203519. doi: 10.1371/journal.pone.0203519 (PMC6136721; doi:10.1371/journal.pone.0203519)
Supplement: S1 Text — (DOC) [file pone.0203519.s002.doc]

Stationarity of the heart rate (or any other physiological quantity) time series over a certain time range is highly complicated issue. In the simplest approach we look at only the mean value of heart rate and demand that the mean values calculated over shorter time segments do not deviate too much from the global mean [1,2]. More strict conditions for stationarity can be formulated by studying time-dependence of both the mean and deviation of the heart rate [3]. These conditions, however, characterize only some very limited aspects of the stationarity of the data. In general, the true stationarity of the system under study means that all background parameters are constant in time. Typically, we can measure or even recognize only part of these parameters. Thus, in practice we are forced to limit our stationarity tests to only few physiologically essential features of the data.

In this study the main focus is in various heart rate variability quantities as spectral powers and fast detrended fluctuations. Since in sleep studies it is more difficult to control the physiological state of the subject than in studies performed in awake conditions we have used a modification of the StatAv stationarity test introduced in refs 1 and 2. In our method all slow oscillations are first removed from the time series using 16^th^ order Butterworth high-pass filter with the cut-off frequency of 0.02 Hz corresponding to the time scale of 50 seconds. The cut-off frequency is set to be so low that this filtering does not affect spectral powers in VLF band. Next the time series is divided into 20 segments of equal length. In each segment the standard deviation is calculated, and after that the distances of the segment deviations from the overall standard deviation are determined. Finally, we calculated the mean of these distances and normalize it with the overall standard deviation. We call this result as StatAvF. If the variability of the data in the terms of deviation is constant over the whole time range StatAvF = 0. If half of the segments have the same non-zero deviation and the rest segments zero deviation, the worst case in our method, StatAvF = 1. Clearly this quantity can be used to characterize the stationarity of the variability (not the mean as StatAv) of the time series, and therefore it is most suitable for measuring stationarity of spectral powers and similar quantities. We used the same limit values of StatAvF as have been used for StatAv [2]: if StatAvF < 0.3 the data is stationary, 0.3 < StatAvF < 0.5 moderately stationary, and StatAvF > 0.5 non-stationary.

**References**

[1] S. M. Pincus, T. R. Cummins, G. G. Haddad, Heart rate control in normal and aborted-SIDS infants, Am. J. Physiol. 264 (Regulatory Integrative Comp. Physiol.) R638-R646, 1993

[2] J. A.Palazzolo, F. G. Estafanous, P. A. Murray, Entropy measures of heart rate variation

in conscious dogs, Am. J. Phys. 274 (Heart. Circ. Physiol.) 43, H1099-H1105, 1998

[3] A. Porta, G. D. Addio, D.Lucini, M. Pagani, Testing the presence of non-stationarities in short heart rate variability series, Computers in Cardiology, 31, 645-648, 2004
