# Supplementary material for: Alterations in heart rate variability in patients with peripheral arterial disease requiring surgical revascularization have limited association with postoperative major adverse cardiovascular and cerebrovascular events
Source: PLoS One. 2018 Sep 13;13(9):e0203519. doi: 10.1371/journal.pone.0203519 (PMC6136721; doi:10.1371/journal.pone.0203519)
Supplement: S1 Table — (DOC) [file pone.0203519.s003.doc]

**Table 8. HRV characteristics in all sleep stages combined according to severity of OSA and occurrence of MACCE.**

|  | AHI <10  n = 18 | AHI 10-20  n = 21 | AHI 20-30  n = 14 | AHI ≥30  n = 21 | No MACCE  n = 52 | MACCE  n = 22 |
| --- | --- | --- | --- | --- | --- | --- |
| HR, 1/min | 68 (59-78) | 58† (50*-67†) | 63 (55-72) | 63 (52-74) | 62 (53-72) | 63 (55-73) |
| NNI Min, ms | 767 (124) | 891 (126) † | 830 (134) | 810 (112) | 828 (127) | 824 (139) |
| NNI Max, ms | 1011 (195) | 1198 (163) * | 1085 (174) | 1157 (252) | 1129 (195) | 1096 (245) |
| NNI Mean, ms | 884 (158) | 1040 (141) † | 958 (159) | 960 (140) | 970 (150) | 950 (173) |
| NNI Dev, ms | 25 [16] | 47 [24] * | 32 [17] | 38 [36] * | 39 [30] | 27 [28] |
| NNI RMSSD, ms | 21 [14] | 31 [19] | 21 [14] | 31 [28] * | 27 [22] | 25 [23] |
| NNI pNN50, % | 2.0 [6.2] | 6.5 [13.6] | 1.1 [6.3] | 5.9 [14.1] | 4.7 [14.0] | 4.2 [6.6] |
| NNI SampEn | 1.3 (0.3) | 1.5 (0.2) | 1.3 (0.3) | 1.4 (0.3) | 1.4 (0.3) | 1.3 (0.3) |
| NNIS Total, ms^2^ | 615 [1013] | 2080 [2314] * | 1245 [1737] | 1119 [2603] | 1435 [2687] | 790 [1622] |
| NNIS ULF, ms^2^ | 45 [138] | 101 [232] | 124 [210] | 72 [65] | 90 [185] | 45 [77] |
| NNIS VLF, ms^2^ | 312 [541] | 1435 [1200] * | 722 [883] | 625 [1195] | 763 [1221] | 408 [1201] |
| NNIS LF, ms^2^ | 153 [252] | 465 [750] * | 189 [366] | 226 [785] | 312 [774] | 114 [275] ‡ |
| NNIS HF, ms^2^ | 69 [71] | 249 [378] * | 63 [140] | 164 [384] * | 150 [318] | 68 [142] ‡ |
| NNIS LF/HF | 2.9 [5.6] | 2.5 [3.5] | 3.3 [3.1] | 1.4 [1.2] | 2.3 [3.7] | 2.1 [2.6] |
| NNIS nLF, nu | 62 (23) | 60 (19) | 65 (16) | 53 (19) | 60 (19) | 59 (23) |
| NNIS nHF, nu | 35 (22) | 38 (18) | 31 (14) | 42 (14) | 38 (17) | 36 (18) |
| NNI Alpha 1 | 1.09 (0.33) | 1.07 (0.29) | 1.06 (0.35) | 0.93 (0.30) | 1.03 (0.29) | 1.03 (0.39) |

Data are mean (standard deviation) or median [interquartile range] except for mean (range) for heart rate (HR). AHI = Apnea-hypopnea index, Alpha 1 = Fractal scaling exponent alpha 1, Dev = Standard deviation of NNI, HF = Power in the high frequency range (0.15-0.4 Hz), LF = Power in low frequency range 0.04-0.15 Hz), MACCE = Major adverse cardiovascular and cerebrovascular event, ms = millisecond, nHF = normalized HF ratio, nLF = normalized LF ratio, NNI = normal-to-normal interval (i.e. time between normal beats in the electrocardiogram), NNIS = NNI spectrum, pNN50 = Proportion of NNI >50 ms, RMSSD = Root mean square of the sum of successive differences (between adjacent normal-to-normal intervals, i.e. beat-to-beat variability), SampEn = Sample entropy, Total = Total power of the NNI spectrum (i.e. overall autonomic activity), ULF = Power in the ultra-low frequency range (≤0.003 Hz), VLF = Power in the very low frequency range (0.003-0.04 Hz). One-way analysis of variance with Dunnett’s post hoc was used to test for differences between each OSA severity group (AHI 10-20/hour, 20-30/hour and >30/hour) vs. AHI <10/hour. Analysis of covariance (ANCOVA) was used to test for independent differences between the OSA severity groups (same as above) and between patients with and without MACCE. The ANCOVA was adjusted for age, body mass index, presence of coronary artery disease and PAD duration. Non-normally distributed variables were log-transformed (natural logarithm) before the statistical analyses (non-transformed values with the width of the IQR in square brackets are shown here for descriptive purpose). *: p<0.05 compared to patients with AHI <10/hour; †: p<0.01 compared to patients with AHI <10/hour (adjusted for age, body mass index, coronary artery disease and duration of peripheral arterial disease); ‡: p<0.05 between patients with and without MACCE (unadjusted analysis, no significant difference after adjustment).
